# To Study the Prevalence of Premalignancies in Teenagers having Betel, Gutkha, Khaini, Tobacco Chewing, Beedi and Ganja Smoking Habit and Their Association with Social Class and Education Status

**DOI:** 10.5005/jp-journals-10005-1243

**Published:** 2014-08-29

**Authors:** Vinay Kumar Srivastava

**Affiliations:** Associate Professor, Department of Pedodontics and Preventive Dentistry, Faculty of Dental Sciences, Institute of Medical Sciences, Banaras Hindu University, Varanasi, Uttar Pradesh, India

**Keywords:** Premalignant lesions, Noxious oral addiction habits, Anti-noxious oral habit policy, Social class

## Abstract

Premalignant oral lesions are usually associated with noxious oral addiction habits. These habits are common in both, high as well as low socioeconomic status but education status of parent and patients significantly affects the development of noxious oral addictions. A total of 872 patients (cases and controls) were included in the study. Social class was determined as per modified Prasad’s classification (1970) with price index correction of 2004. Prevalence of lichen planus, to be only 0.4 and 2.6% present in groups III and IV of cases, and submucous fibrosis (SMF) – stromal one lanocytic foci – was 2.4% in male (group III) whereas it was not found in female cases (group IV). Teenagers having higher frequency and longer duration of noxious habits were more prone for development of premalignant lesions. 0.6% of leukoplakia, 0.3% erythroplakia, 0.7% lichen planus and 0.7% submucous fibrosis were present in 872 observed patients of control and cases.

**How to cite this article:** Srivastava VK. To Study the Prevalence of Premalignancies in Teenagers having Betel, Gutkha, Khaini, Tobacco Chewing, Beedi and Ganja Smoking Habit and Their Association with Social Class and Education Status. Int J Clin Pediatr Dent 2014;7(2):86-92.

## INTRODUCTION

There is rise in oral cancer in India. There are many factors which are known to be associated with oral cancer; one of them is noxious oral addiction habits. These noxious oral habits include betel, gutkha, khaini, tobacco chewing, beedi and ganja smoking habits. Many articles advocate that these oral habits related to the origin of oral cancers. These habits are most popular noxious habits in and around holy city Varanasi. It has been seen that low socioeconomic status people are more inclined for these addictions.

It has been seen that teenagers are more inclined toward above noxious oral addiction which is initially started as enjoy and fun. As time passes, they become addicted to the habit. These oral addiction is seen in both high as well as low socioeconomic and education status respectively. Longer duration of exposure to the above noxious substances can lead to reactive changes in the oral mucosa that may have potential to change into malignancies. Those mucosal reactive lesions that have increased chances of turning into malignancies are called premalignant lesions (leukoplakia, erythroplakia, palatal keratosis associated with smoking, lichen planus and submucous fibrosis). This study determines prevalence of premalignancies in teenagers having noxious oral addictions and their correlation with social class and education status.

## SIGNIFICANCE OF THE STUDY

1. To find out the potential individuals for cancer development in coming years and its preventive program.
2. To correlate the effect of socioeconomic and education status of people to the addiction habit.
3. Need of preventive and educative program.

### METHODOLOGY

Ethical committee approval and informed consent were taken from teenager volunteers. This is a case control study and financed by Banaras Hindu University (BHU), Under XI plan head ‘others’ Research grant. The study was conducted on the teenagers (12 to 19 years) with 56.9% males and 43.1% females ratio (872 patients). Cases and controls were divided into four groups: Group I – control male; group II – control female; group III – cases male and group IV – cases female.

The cases included the patients having habits of betel and gutkha, khaini, tobacco chewing, beedi and ganja smoking habit; consulting the dental Outpatient Department (OPD), IMS, BHU, either for follow-up of already diagnosed cases or for current diagnosis of premalignant lesion. Controls were the patients reported to the dental OPD, Institute of Medical Sciences, BHU, with some other ailments but devoid of the above habits. Social class was determined as per modified Prasad’s classification (1970) with price index correction of 2004.

The information was recorded on predesigned questionnaires. Teenagers were interviewed in depth, and data were recorded on self-prepared questionnaires. Data analysis was done by the SPSS version 16 software and following results have been obtained.

## SOCIAL CLASS

Socioeconomic status also influences social security in terms of accessibility, affordability, acceptability and actual utilization of oral and general health. Prasad developed a classi-fication (scale) to measure socioeconomic status, which is based on per capita monthly income. It has been extensively used in the Indian scenario. By 1993 to 1994, the infation rate was governed by the all India whole price index series creating an urgent need to link Prasad’s classification with the all India whole price index. In order to solve this problem, a hypothetical value (0.53) has been developed in relation to base year of 1993 to 1994, when new series of the all India wholesale price index started (AIWPI) (Table 1).^1,2^ It is a simple method of multiplying the income limits of the classi-fication with a multiplication factor and rounding off the values to the nearest rupee. Multiplying the AIWPI at the time of study by the hypothetical value could help to drive the multiplication factor.

Therefore, the multiplication factor = value of AIWPI × 0.53.

                                                     = 198 × 0.53

                                              MF = 104.94

**Graph 1:**
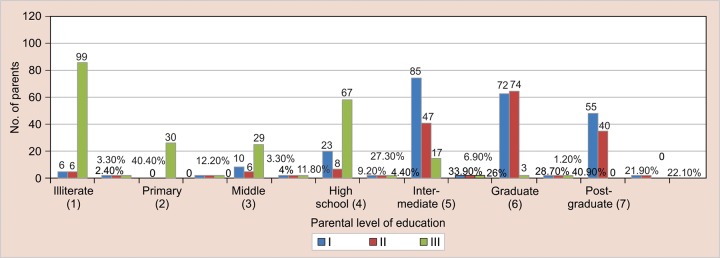
The representation of parent education variables in groups of control and cases

Table 2 and Graph 1 show variables in parent education among control and cases. There were significant difference of education in groups of control and cases (p < 0.001). In group I, only 2.4% parents were illiterate and 84.5% parents were have intermediate and above education within the group. In group II, 3.3% parents were illiterate and 89% parents were having intermediate and above education within the group. In group III, 40.4% parents were illiterate and only 20% parents were having intermediate and above education. In group IV, 61% parents were illiterate and 39% parents were having only primary education.

**Graph 2:**
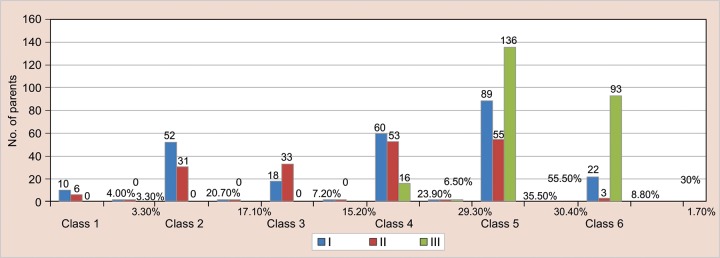
The representation of variables of social class (modified Prasad’s classification) in group of control and cases

Table 3 and Graph 2 show variables of social class among group of control and cases. There were significant difference between control and cases (p = 0.001). In group I, 31.9% family status belonged to class 1 to 3 of social class, 59.4% family status belonged to class 4 to 5 and only 8.8% belonged to class 6, i.e. below poverty line. In group II, 38.6% family status belonged to class 1 to 3 and 59.7% family status belonged to class 4 and 5. Only 1.7% family status belonged to class 6, i.e. below poverty line. In group III, most of the family status beloned to social class 5. Only 6.5% family belonged to class 4. None of the family of group III belonged to social class 1, 2 and 3. In group IV, most of the family belong to social class 6, i.e. 90.8 and 9.2% belonged to social class 5.

Table 4 shows habit one in control and cases. There were significant difference in habit one of control and cases (p < 0.001). Habit one present in 50.2% of group III individuals, and habit one did not present in groups I, II and I V.

There were significant difference in habit two of control and cases (p < 0.001). Habit two present in 40% of group III individuals and 100% individuals of group IV have habit two.

There were significant difference in habit three of control and cases (p < 0.001). Habit three present in 44.4% of individuals. Habit three absent in groups I, II and I V.

**Table 1:** **Table 1:** Prasad’s classification per capita monthly income (Jan 2012)

*S. no.*		*Social class*		*Prasad’s classification^2^ per capita monthly income (1961)*		*Modified Prasad’s classification^1^ per capita monthly income (2012 Jan)*	
1		Upper high		100 and above		10,500 or more	
2		High		50-99		5250-10390	
3		Upper middle		30-49		3150-5145	
4		Lower middle		15-29		1575-3045	
5		Poor		Below 15		<1575	
6		Very poor or below poverty line (BPL)		―		<525	

**Table 2:** **Table 2:** Parent and patient education variables in groups of control and cases

*Parent education*		*I*		*II*		*III*		*IV*		*Total*			
Illiterate (1)		6 (2.4%)		6 (3.3%)		99 (40.4%)		119 (61%)		230 (26.4%)		χ*^2^ 829.89, df 18,* *p < 0.001*	
Primary (2)		0		0		30 (12.2%)		76 (39%)		106 (12.2%)			
Middle (3)		10 (4%)		6 (3.3%)		29 (11.8%)		0		45 (5.2%)			
High school (4)		23 (9.2%)		8 (4.4%)		67 (27.3%)		0		98 (11.2%)			
Intermediate (5)		85 (33.9%)		47 (26%)		17 (6.9%)		0		149 (17.1%)			
Graduate (6)		72 (28.7%)		74 (40.9%)		3 (1.2%)		0		149 (17.1%)			
Postgraduate (7)		55 (21.9%)		40 (22.1%)		0		0		95 (10.9%)			
*Patient education*													
Illiterate (1)		3 (1.2%)		3 (1.7%)		42 (17.1%)		104 (53.3%)		152 (17.4%)		*χ^2^ 801.52, df 12, p < 0.001*	
Primary (2)		0		1 (0.6%)		70 (28.6%)		82 (42.1%)		153 (17.5%)			
Middle (3)		34 (13.5%)		12 (6.6%)		87 (35.5%)		8 (4.1%)		141 (16.2%)			
High school (4)		39 (15.5%)		25 (13.8%)		45 (18.4%)		1 (0.5%)		110 (12.6%)			
Intermediate (5)		175 (69.7%)		140 (77.3%)		1 (0.4%)		0		316 (36.2%)			
Total		251 (28.8%)		181 (20.8%)		245 (28.1%)		195 (22.4%)		872 (100%)			

There were significant difference in habit four of control and cases (p < 0.001). Habit four present in 30.6% of group III individuals and 4.1% of group IV individuals.

There were significant difference in habit five of control and cases (p < 0.001). Habit five present in 1.6% of group III individuals and 0.5% of group IV individuals.

Table 5 shows variables of frequency and duration of habits in groups of control and cases. There were significant difference in variables of frequency one in groups of control and cases (p < 0.001). Group III was having 52.7% frequency one (i.e. > 2 times/day of habits) and 47.3% frequency two (i.e. < 2 times/day). Group IV was having frequency one and 50.3% having frequency two (< 2 times/day).

A total of 32.7% individuals having duration one (i.e. > 3 years of consumption) and 67.3% individuals having frequency two (i.e. < 3 years of consumption) in group III. 28.2% individuals have duration one (i.e. > 3 years of consumption) and 71.8% individuals have duration two (i.e. < 3 years of consumption) in group I V.

Table 6 shows variables of lesions in control and cases. Lesion one variables are not significant in groups of control and cases (p = 0.005). Lesion one (leukoplakia) absent in groups I, II and IV, while only 2% lesion one present in groups III.

Lesion two (erythroplakia) absent in groups I, II and I V, while only 0.3% lesion two (erythroplakia) present in group III. Nonsignificant difference was present in control and cases (p = 0.053).

**Table 3:** **Table 3:** Variables of social class (modified Prasad’s classification) in group of control and cases

*Variables of social class*		*I*		*II*		*III*		*IV*		*Total*			
Class 1		10 (4.0%)		6 (3.3%)		0		0		16 (1.8%)		*χ^2^ 615.49* *df 15, p < 0.001*	
Class 2		52 (20.7%)		31 (17.1%)		0		0		83 (9.5%)			
Class 3		18 (7.2%)		33 (18.2%)		0		0		51 (5.8%)			
Class 4		60 (23.9%)		53 (29.3%)		16 (6.5%)		0		129 (14.8%)			
Class 5		89 (35.5%)		55 (30.4%)		136 (55.5%)		18 (9.2%)		298 (34.2%)			
Class 6		22 (8.8%)		3 (1.7%)		93 (38%)		177 (90.8%)		295 (33.8%)			
Total		251 (28.8%)		181 (20.8%)		245 (28.1%)		195 (22.4%)		872 (100%)			

**Table 4:** **Table 4:** Variables of habits in groups of control and cases

*Habits*		*I*		*II*		*III*		*IV*		*Total*			
*Habit 1 (pan, tobacco, smoking and gutkha)*													
• 1 (present)		0		0		123 (50.2%)		0		123 (14.1%)		χ^2^ 366.47,	
• 2 (absent)		251 (100%)		181 (100%)		122 (49.8%)		195 (100%)		749 (85.9%)		df 3, p < 0.001	
*Habit 2 (pan, gutkha without tobacco and smoking)*													
• 1 (present)		0		0		98 (40%)		195 (100%)		293 (33.6%)		χ^2^ 608.45,	
• 2 (absent)		251 (100%)		181 (100%)		147 (60%)		0		579 (66.4%)		df 3, p < 0.001	
*Habit 3 [tobacco (khaini), smoking]*													
• 1 (present)		0		0		109 (44.5%)		0		109 (12.5%)		χ^2^ 318.80,	
• 2 (absent)		251 (100%)		181 (100%)		136 (55.5%)		195 (100%)		763 (87.5%)		df 3, p < 0.001	
*Habit 4 (ganja smoking, pan)*													
• 1 (present)		0		0		75 (30.6%)		8 (4.1%)		83 (9.5%)		χ^2^ 178.66,	
• 2 (absent)		251 (100%)		181 (100%)		170 (69.4%)		187 (95.5%)		789 (90.5%)		df 3, p < 0.001	
*Habit 5 (other habits–nail biting)*													
• 1 (present)		0		0		4 (1.6%)		0		4 (0.5%)		χ^2^ 10.284,	
• 2 (absent)		251 (100%)		181 (100%)		241 (98.4%)		195 (100%)		868 (99.5%)		df 3, p > 0.001	
Total		251 (28.8%)		181 (20.8%)		245 (28.1%)		195 (22.4%)		872 (100%)			

**Table 5:** **Table 5:** Variables of frequency and duration of habits among control and cases

*Variables of frequency*		*I*		*II*		*III*		*IV*		*Total*			
Frequency 1 (>2 times/day)		0		0		129 (52.7%)		97 (49.7%)		226 (25.9%)		χ^2^ 872.79, df 06, p < 0.001	
Frequency 2 (<2 times/day)		0		0		116 (47.3%)		98 (50.3%)		214 (24.5%)		N = 872	
Frequency 3 (nonconsuming)		251 (100%)		181 (100%)		0		0		432 (49.5%)		N = 872	
*Variables in duration* Duration 1 (>3 years of consumption)		0		0		80 (32.7%)		55 (28.2%)		135 (15.5%)		χ^2^ 874.002, df 06, p < 0.001	
Duration 2 (<3 years of consumption)		0		0		165 (67.3%)		140 (71.8%)		305 (35%)		N = 872	
Duration 3 (nonconsuming)		251 (100%)		181 (100%)		0		0		432 (49.5%)		N = 872	
Total		251 (28.8%)		181 (20.8%)		245 (28.1%)		195 (22.4%)		872 (100%)			

Lesion four (lichen planus) absent in groups I and II. Only 0.4 and 2.6% present in groups III and IV of cases. Lesional variables in control and cases were nonsignificant (p = 0.004).

In lesion five (submucous fibrosis), there were significant differences in control and cases (p = 0.001). Only group III has 2.4% of lesion five. And, rest of the group did not show the presence of lesion five (i.e. groups I, II and IV).

**Table 6:** **Table 6:** Variables of premalignant lesions in groups of control and cases

*Lesions*		*I*		*II*		*III*		*IV*		*Total*			
*Lesion 1 (leukoplakia)*													
• 1 (absent)		251 (100%)		181 (100%)		240 (98%)		195 (100%)		867 (99.4%)		χ^2^ 12.87,	
• 2 (present)		0		0		5 (2%)		0		5 (0.6%)		df 3, p = 0.005	
*Lesion 2 (erythroplakia)*													
• 1 (absent)		251 (100%)		181 (100%)		242 (98.8%)		195 (100%)		869 (99.7%)		χ^2^ 7.70,	
• 2 (present)		0		0		3 (0.3%)		0		3 (0.3%)		df 3, p = 0.053	
*Lesion 3 (palatal keratosis associated with reverse smoking)*													
• 1 (absent)		251 (100%)		181 (100%)		245 (100%)		195 (100%)		872 (100%)		N = 872	
• 2 (present)		0		0		0		0		0			
*Lesion 4 (lichen planus)*													
• 1 (absent)		251 (100%)		181 (100%)		244 (99.6%)		190 (97.4%)		866 (99.3%)		χ^2^ 13.317,	
• 2 (present)		0		0		1 (0.4%)		5 (2.6%)		6 (0.7%)		df 3, p = 0.004	
*Lesion 5 (submucous fibrosis)*													
• 1 (absent)		251 (100%)		181 (100%)		239 (97.6%)		195 (100%)		866 (99.3%)		χ^2^ 15.46,	
• 2 (present)		0		0		6 (2.4%)		0		6 (0.7%)		df 3, p = 0.001	
Total		251 (28.8%)		181 (20.8%)		245 (28.1%)		195 (22.4%)		872 (100%)			

## DISCUSSION

*Lesion one or leukoplakia*: Oral leukoplakia is a white patch that cannot be rubbed off and cannot be characterized clinically or histopathologically as any other condition, and is not associated with any physical or chemical agent except tobacco. Usually, term leukoplakia implies only the clinical feature of a persistent adherent white patch.

A typical white patch of leukoplakia develops slowly, over weeks to months. The lesion may eventually become rough in texture, and sensitive to touch, heat, spicy foods and other irritation.

In our study, lesion one, i.e. leukoplakia variables, is not significant in groups of control and cases (p = 0.005). Lesion one (leukoplakia) absent in groups I, II and IV, while only 2% lesion one present in group III. Males are more prone for the development of leukoplakia than females. This is probably males having more frequency and duration of tobacco chewing than females.

Erythroplakia is a clinical term used to describe a fery red patch that cannot be clinically or pathologically distinguished a reasonable other definable disease.^3^ The clinical appearance of erythroplakia is described as red macule or patch with a soft, velvety texture most often occurring on foor of mouth, lateral tongue, retromolar pad and soft palate.^4^

Our results indicate that tobacco chewing is a strong risk factor for erythroplakia. Dose response was also correlated with frequency of noxious habits with risk of erythroplakia.

Group III has been reported 0.3% prevalence of erythroplakia as a risk factor for both oral leukoplakia and oral cancer. Groups I, II and III did not report cases of erythroplakia.

Our results indicate that group III has a strong risk factor for development of erythroplakia as comparison groups I, II and IV.

Chewing tobacco may possibly be a strong risk factor for erythroplakia than smoking in the Indian population as is suggested for oral leukoplakia.^5^ Chewing tobacco may have a stronger effect than smoking because of direct contact of the tobacco carcinogens with the oral epithelium as chewing tobacco kept in the oral cavity.

*Reverse smoking*: None of the cases found as having tendency of reverse smoking in teenagers.

*Lichen planus*: It is a papulosquamous disease that characteristically involves mucous membrane, especially oral mucosa, genital mucosa as well as skin. The prevalence of lichen planus is unknown; however, it is thought to be less than 1% of the population.^6^

Our study finds prevalence of lichen planus to be only 0.4 and 2.6% present in groups III and IV of cases, among patient attending Dental OPD of IMS, BHU, Varanasi, and outside the OPD. In control group, i.e. groups I and II, no lesions of lichen planus were seen, which suggest that noxious oral habits have a definite relation with development of premalignant lichen planus.

It has also been seen that groups III and IV have lower socioeconomic status than groups I and II. Cases, i.e. socioeconomic status of group I V, are lower than groups III, II and I. Therefore, socioeconomic status of family also plays an important role in the development of the noxious habits.

In group I, only 2.4% parents were illiterate and 84.5% parents were have intermediate and above education within the group. In group II, 3.3% parents were illiterate and 89% parents were having intermediate and above education within the group. In group III, 40.4% parents were illiterate and only 20% parents were having intermediate and above education. In group I V, 61% parents were illiterate and 39% parents were having only primary education. Parental education also has affect over the development of noxious oral habits. It has been seen that higher parental education prevents development of noxious habits in their offspring.

Illiteracy or lower education status encourages development of noxious habits. In group I, only 1.2% patients were illiterate and 98.7% patients were having middle and above education. In group II, only 1.7% patients were illiterate and 98.3% patients were having education between middle and intermediate. In group III, 17.1% patients were illiterate and 28.6% were having primary education. 35.5% having middle and 18.4% high school, and only 0.4% having education of intermediate. In group IV, 53.3% were illiterate and 42.1% patients were having primary education. Only 4.6% patients were having middle and high school education. None of the patient were having intermediate or above education in group IV.

Recent studies reveal that age of lichen planus patient ranged from 10 to 65 years, and majority of patient falls in age group of 21 to 50 years. This finding supports the suggestion that younger patients tend to be affected in tropical countries.^7^ In our study, only teenagers were participated.

Lichen planus is said to affect female preferentially, however, in our study, 0.4% male case and 2.6% female case were affected. Male:female ratio is 0.153 recorded, has refects no significant difference in sex distribution (p = 0.004).

It is reported that childhood lichen planus is rare^8^ and familial lichen planus is not reported. One study reveals that incidence of childhood lichen planus is 2% of total lichen planus. Our study reveals that 2.6% female teenagers were affected. Study reveals that isolated oral lichen planus present in 15 to 35% of lichen planus patient.^9,10^

Lichen planus tends to be intensely pruritic when purities present. It ranges from mild irritation to severe itching. In oral lichen planus, patient complains of burning and itching.

In our study, the incidence of lichen planus was 0.4% in male and 2.6% in female of cases (groups III and IV) which is comparable to the study of tag-El-Din Anbar et al [Dermatology. Online journal 2003;11(2):4] who find 0.28% incidence.

*Oral submucous fibrosis*: It is chronic, complex irreversible, highly potent, premalignant condition characterized by juxtaepithelial inflammatory reaction and progressive fibrosis of the submucosal tissues.

As the disease progresses, the jaw become rigid to the point that the sufferer feels ristricted mouth opening.^6^ Chronic exposure to the betel nut (gutkha, arecanut) or pan (betel quid chewing), chilli, pepper and prolong deficiency of iron and zink may lead to the alteration in oral mucosa which causes hypersensitivity to these irritants. This hypersensitivity reaction may often result in formation of collagen fibers in lamina propria. These collagen fibers are nondegradable and believed to be involved in the pathogenesis of submucous fibrosis.^11-13^

In initial phase of the disease, the mucosa feels leathery with palpable vertical and circular fibrous band. In advanced stage, the buccal mucosa lost its resiliency and become blanched and stiff. It is postulated that disease developed initially in posterior part of oral cavity and gradually spread toward lips.

Since, pan masala and gutkha have higher concentration of areca nut and appeared to cause disease.

In our study, 2.4% males (group III) were affected and SMF did not find in females cases (group IV).

*Mortality/morbidity*: Submucous fibrosis (SMF) has high rate of morbidity because it causes a progressive inability to open the mouth, resulting difficulty in eating and consequent nutritional deficiency. SMF has significant mortality because it transform into oral cancer, specially squamous cell carcinoma at a rate of 7.6%.

Since, parental education, patient education and family economic status have a significantly affect in the development of noxious habits. These noxious habits have been responsible for the development of premalignant oral lesions.

This is the first time that a strong association between noxious oral habit and relative risk of leukoplakia, erythroplakia, palatal keratosis, lichen planus, submucous fibrosis and socioeconomic status was found in our case control study.

## CONCLUSION

Based on the present case control study, the following may be concluded.

- Lesion one (leukoplakia) absent in groups I, II and IV, while only 2% lesion one present in group III. Group III has been reported 0.3% prevalence of erythroplakia of as a risk factor for both oral leukoplakia and oral cancer. Groups I, II and III did not report cases of erythroplakia. In our study, finds prevalence of lichen planus to be only 0.4 and 2.6% present in groups III and IV of cases. In our study, prevalence of SMF was 2.4% in male (group III) and SMF did not found in female cases (group IV).
- Since, parental education, patient education and family economic status have a significantly affect in the development of noxious habits. These noxious habits seem to be responsible for the development of premalignant oral lesions.
- Teenagers having higher frequency and longer duration of noxious habit were more prone for development of premalignant lesions.
- Around 0.6% of leukoplakia, 0.3% erythroplakia, 0.7% lichen planus and 0.7% submucous fibrosis were present in 872 observed patients of control and cases.

## RECOMMENDATIONS

There is a need for a national program on dental health in the country that should be integrated with community-based health program which would have both informative and educative about the noxious oral habits and their related risk factors in the development of oral cancerous lesions, enabling an individual to increase in knowledge and influencing the attitude, so as to bring out a qualitative change in the lifestyle and behavior of the population. Following point should be kept in mind at the time of anti-noxious oral habit policy (ANOHP) development.

- To regret noxious oral habits in the teenagers, we should encourage school to develop policies, to provide a more supportive environment, to drop the noxious habits by showing lesional photograph of oral cancers and related risk of noxious habits. The advantages of school health education are cost-effective, continuity in instructions and access to all at one time. Active involvement is key to effective learning and, hence, classroom-based oral health education as well as regular group meetings with their parents are recommended.
- To educate teenagers about risk factor for development of cancerous lesion, of having betel, gutkha, khaini, tobacco chewing, beedi and ganja smoking habit.
- Teenagers should be benefited from information on oral health education, keeping in mind that they represent a challenging group as they are on the verge of establishing their independence from parental influence. Acquiring correct knowledge about related risk factor of noxious oral habits. Healthful knowledge is capable of influencing our attitude and both are instrumental in reducing the disease burden as well as serve as an eye opener to the facts that predispose the individual and community to the disease.
